# Endothelial Cell-Derived PCSK9 in Atherosclerosis: Pathophysiological Roles and Therapeutic Perspectives

**DOI:** 10.31083/RCM44885

**Published:** 2025-10-21

**Authors:** Pei Wang, Haixia Wang, Dongdong Yan, Zheng Zhang

**Affiliations:** ^1^The First Clinical Medical College, Lanzhou University, 730000 Lanzhou, Gansu, China; ^2^Department of Cardiology, The First Hospital of Lanzhou University, 730000 Lanzhou, Gansu, China; ^3^Gansu Provincial Clinical Research Center for Cardiovascular Diseases, 730000 Lanzhou, Gansu, China

**Keywords:** endothelial cells, subtilisin/kexin type 9, atherosclerosis

## Abstract

Atherosclerosis (AS), the primary pathological basis for cardiovascular disease (CVD), is initiated by endothelial dysfunction. This review aimed to summarize the current understanding of endothelial cell-derived proprotein convertase subtilisin/kexin type 9 (PCSK9) in the pathogenesis of AS and to explore the potential of using PCSK9 as a therapeutic target. Endothelial PCSK9 contributes to AS progression by regulating lipid metabolism through low-density lipoprotein receptor (LDLR) degradation and promoting inflammatory responses, oxidative stress, endothelial apoptosis, and increased vascular permeability. Recent evidence indicates that endothelial-derived PCSK9 is upregulated under pathological conditions and exerts multiple atherogenic effects independent of circulating PCSK9. Experimental studies have demonstrated that silencing or inhibiting endothelial PCSK9 alleviates endothelial dysfunction, reduces plaque development, and mitigates inflammatory responses. Moreover, PCSK9 may modulate the redox balancing and cellular signaling pathways involved in vascular homeostasis. Endothelial PCSK9 plays a critical role in the initiation and progression of AS through mechanisms beyond lipid regulation. Targeting endothelial PCSK9 may represent a novel and promising strategy for preventing and treating AS, warranting further preclinical and clinical investigation.

## 1. Introduction

Atherosclerosis (AS) serves as the primary pathological foundation of cardiovascular disease (CVD), and its progression is characterized by vascular endothelial cells (ECs) dysfunction, dyslipidemia, inflammation, and other contributing factors, with ECs dysfunction initiating the development of AS. The onset of AS is triggered by endothelial dysfunction and injury [1]. ECs form a continuous monolayer of flattened cells that line the luminal surface of the vasculature throughout the circulatory system, from the heart to the smallest microvessels, thereby serving as a barrier between plasma and the vascular wall tissue [2]. ECs are metabolically active and multifunctional, contributing to the maintenance of internal homeostasis, the regulation of normal blood flow, and the preservation of vascular patency. However, disruption of the endothelial barrier facilitates lipid retention, monocyte adhesion, and transmigration into the vascular wall, which in turn triggers localized inflammatory responses and initiates atheromatous plaque formation [3]. Given the central role of endothelial integrity in vascular homeostasis, strategies aimed at preserving endothelial function have emerged as a major research focus in the prevention and treatment of AS.

The preprotein convertase subtilisin/kexin type 9 (PCSK9) is a serine protease secreted by the liver with high hepatic expression, which elevates plasma low-density lipoprotein cholesterol (LDL-C) by promoting the degradation of the low-density lipoprotein receptor (LDLR) in the circulation [4]. Based on this mechanism of action, PCSK9 has been identified as a key therapeutic target for lipid-lowering interventions. For instance, the clinical use of PCSK9 inhibitors—such as the monoclonal antibodies evolocumab and alirocumab—has been shown to significantly lower LDL-C levels and improve cardiovascular outcomes [5]. However, PCSK9 is expressed in many tissues other than the liver, including the small intestine, kidney, brain tissue, and blood vessel wall cells [6]. Most current research and clinical applications of PCSK9 have primarily focused on its hepatic origin within the circulatory system, while limited attention has been given to PCSK9 that is endogenously expressed by vascular ECs [7]. It has been noted that human umbilical vein ECs (HUVECs) barely express PCSK9 in the physiological state, but can be induced to synthesize and secrete PCSK9 in response to inflammatory stimuli [8]. Another study found that ECs localized in atherosclerotic lesions can secrete and form higher concentrations of PCSK9 locally in blood vessels [9]. These findings suggest that PCSK9 derived from ECs may be directly involved in the formation and progression of atherosclerotic plaques, and that its biological effects may occur through mechanisms independent of conventional lipid-lowering pathways. This review summarizes recent mechanistic insights and research advances concerning the role of endothelial PCSK9 in AS, with the aim of addressing current knowledge gaps and exploring potential therapeutic strategies targeting endothelial PCSK9.

## 2. Biological Function and Mechanism of Action of PCSK9

PCSK9 is a member of the prealbumin convertase Bacillus subtilis protease family discovered in 2003 and consists of a 74 kDa-sized zymogen protein comprising 692 amino acids, including a signal peptide, a prodomain, a catalytic domain, and a C-terminal cysteine/histidine-rich structural domain [10]. PCSK9 is activated by autocatalytic cleavage within the endoplasmic reticulum of hepatocytes prior to its secretion into the circulation; however, it does not possess conventional proteolytic enzymatic activity. Instead, PCSK9 binds with high affinity to LDLR on the surface of hepatocytes, leading to the formation of a PCSK9–LDLR complex that facilitates lysosomal degradation of the receptor and inhibits its recycling. This process results in a reduced density of LDLRs on the hepatocyte surface, thereby diminishing LDL clearance and subsequently increasing plasma LDL-C concentrations [11]. The *PCSK9* gene is located on human chromosome 1p32.3, and its mutations are generally classified into two categories: gain-of-function (GOF) and loss-of-function (LOF). Genetic studies have demonstrated that GOF mutations in the *PCSK9* gene enhance the degradation of LDLR, thereby leading to hypercholesterolemia and elevated cardiovascular risk, whereas LOF mutations markedly reduce LDL-C levels by more than 40% and confer cardiovascular protection [12].

**Fig. 1.**
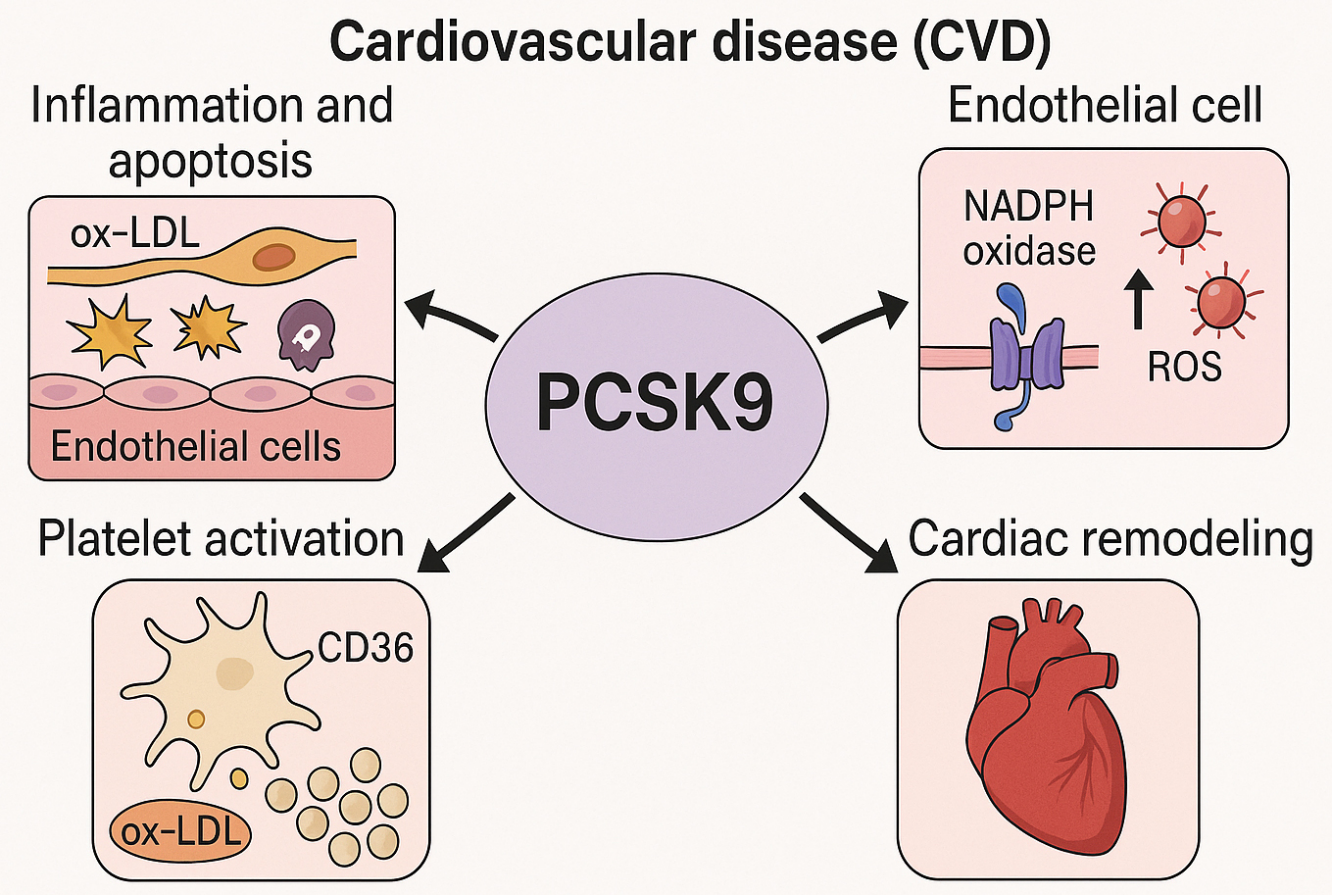
**Mechanistic insights into the non-lipid cardiovascular effects of PCSK9**. This schematic depicts the multifaceted roles of PCSK9 in the pathogenesis of CVD, extending beyond its canonical involvement in lipid regulation. PCSK9 has been shown to promote endothelial inflammation and apoptosis in response to oxidized LDL (ox-LDL), enhance ROS generation via NADPH oxidase activation, facilitate platelet activation through CD36-mediated ox-LDL uptake and downstream signaling pathways (e.g., Src/MAPK), and contribute to concentric cardiac remodeling. These mechanisms highlight PCSK9 as a pleiotropic modulator in the progression of CVD. PCSK9, proprotein convertase subtilisin/kexin type 9; CVD, cardiovascular disease; ROS, reactive oxygen species; NADPH, nicotinamide adenine dinucleotide phosphate; Src/MAPK, sarcoma/mitogen-activated protein kinase. The figure was created with BioRender.

PCSK9 is not essentially a protease in the traditional sense, and its activity is mainly manifested as a secreted factor interacting with a variety of receptors; thus, in addition to its role in cholesterol metabolism, PCSK9 has pleiotropic properties. Recent studies have shown that PCSK9 may influence various cardiovascular conditions by affecting inflammatory responses, oxidative stress, endothelial function, platelet activation, and myocardial remodeling [13]. In inflammatory pathways, PCSK9 has been identified as a pro-inflammatory mediator interacting directly with leukocytes through novel receptors such as cyclase-associated protein 1 (CAP1). Activation of CAP1 by PCSK9 triggers intracellular signaling via the spleen tyrosine kinase/protein kinase C-δ (Syk/PKC-δ) pathway, upregulating Toll-like receptor 4 (TLR4) expression and nuclear factor kappa B (NF-κB) pathway activation, thereby increasing pro-inflammatory cytokines and exacerbating vascular inflammation [14]. Supporting these findings, PCSK9-knockout mice display reduced inflammatory responses to endotoxins, suggesting that PCSK9 promotes inflammation independently of LDLR pathways; PCSK9 also exacerbates oxidative stress and endothelial dysfunction. Recent studies indicate that under pro-atherogenic conditions such as exposure to oxidized LDL or a high-fat environment, endothelial PCSK9 enhances the activation of nicotinamide adenine dinucleotide phosphate (NADPH) oxidase enzymes, particularly NOX2, via redox-sensitive signaling pathways (e.g., p38 mitogen-activated protein kinase [p38 MAPK]), thereby promoting increased reactive oxygen species (ROS) generation [15]. Elevated ROS not only damages ECs directly, inducing apoptosis, but also further oxidizes LDL, perpetuating inflammation and AS progression [16]; Moreover, PCSK9 has direct implications in platelet activation and thrombosis. Circulating PCSK9 binds specifically to cluster of differentiation 36 (CD36) on platelet membranes, initiating a series of intracellular signaling events involving Src-family kinases and MAPK pathways. These events lead to increased platelet aggregation and granule release, intensifying thrombotic risk. Importantly, inhibition or genetic deletion of CD36 abrogates these pro-thrombotic effects, highlighting the specificity and clinical relevance of the PCSK9-CD36 interaction [17]. A clinical study reported that inhibition of PCSK9 significantly reduced biomarkers of platelet activation and endothelial dysfunction in patients with acute coronary syndrome (ACS). The same study demonstrated that PCSK9 was associated with platelets and vascular ECs in left internal mammary artery (LIMA) segments, and its inhibition attenuated this interaction [18]; In addition, PCSK9 influences myocardial metabolism and cardiac remodeling. Genetic deletion of PCSK9 in mouse models results in concentric cardiac remodeling characterized by increased left ventricular wall thickness and cardiac mass, despite normal ejection fraction. This cardiac phenotype appears linked to myocardial lipid accumulation due to upregulated LDLR and CD36 expression, which increases lipid uptake into cardiomyocytes [19]. Consistent with animal findings, human studies demonstrate increased left ventricular mass in carriers of PCSK9 LOF variants, although systolic function remains unaffected, further illustrating the complex physiological roles of PCSK9 beyond lipid metabolism (Fig. 1).

In conclusion, PCSK9 has traditionally been recognized for mediating LDLR degradation and regulating cholesterol metabolism, but its functional scope within the cardiovascular system has recently been expanded to encompass pro-inflammatory activities and modulation of vascular cell function. These pleiotropic effects may account for the observed cardiovascular benefits of PCSK9 inhibition that extend beyond cholesterol-lowering alone. Therefore, the biological functions and underlying mechanisms of PCSK9 in vascular tissues, particularly within ECs, warrant further in-depth investigation.

## 3. The Key Role of ECs in AS

A monolayer of ECs forms the vascular endothelium, which is essential for maintaining vascular homeostasis by regulating vascular permeability, releasing vasoactive substances, and inhibiting blood coagulation. When risk factors such as hyperlipidemia, cigarette smoking, and hypertension affect the vasculature, ECs, which serve as the primary barrier, become injured, and their subsequent dysfunction is considered the initiating event in the development of AS [20]. EC dysfunction is primarily characterized by diminished nitric oxide (NO) bioavailability, increased production of ROS, and upregulated expression of inflammatory mediators and adhesion molecules, all of which contribute to arterial inflammation and atherogenic plaque development [16].

In the early stages of AS, EC injury leads to increased vascular permeability, thereby facilitating the infiltration of lipids into the subendothelial space. Subsequently, ECs upregulate the secretion of chemokines (e.g., monocyte chemoattractant protein-1 [MCP-1]) and adhesion molecules (e.g., vascular cell adhesion molecule-1 [VCAM-1], intercellular adhesion molecule-1 [ICAM-1]), which facilitate monocyte adhesion, transmigration, and differentiation into macrophages. These macrophages internalize oxidized LDL (ox-LDL), giving rise to foam cell formation and lipid plaque accumulation [21]. Concurrently, ECs exposed to ox-LDL and inflammatory mediators may undergo apoptosis or detachment, resulting in disruption of endothelial integrity and exposure of the plaque surface, which subsequently elevates the risk of thrombosis [22]. In summary, a healthy endothelium is characterized by anti-adhesive, anticoagulant, and vasoregulatory properties, whereas in AS, it acquires an activated phenotype that contributes to and amplifies local inflammation. An imbalance between endothelial injury and repair is sustained throughout the progression of AS.

Overall, EC dysfunction plays a central role in both the initiation and progression of AS, and factors that exacerbate inflammation, apoptosis, or EC impairment can accelerate plaque development. PCSK9 has emerged as a novel regulator of EC function, and its regulatory mechanisms and specific roles within the endothelium warrant further investigation to elucidate its contribution to AS.

## 4. Expression and Regulation of PCSK9 in ECs

PCSK9 is primarily produced and secreted into the circulation by organs such as the liver and small intestine, whereas its expression in vascular ECs and immune cells is relatively low under physiological conditions. However, PCSK9 expression is markedly upregulated in ECs under inflammatory and atherosclerotic conditions. Animal studies have demonstrated substantially elevated PCSK9 levels in arterial plaques of high-fat diet-fed ApoE^-⁣/-^ mice compared to normal controls, whereas PCSK9-positive signals were barely detectable in the normal arterial intima, suggesting local synthesis and accumulation by ECs within atherosclerotic lesions [23].

Endothelial PCSK9 expression has been shown to be upregulated by multiple AS-associated stimuli, including oxidized low-density lipoprotein (ox-LDL), proinflammatory cytokines (e.g., tumor necrosis factor-α [TNF-α], interleukin-1β [IL-1β]), lipopolysaccharide (LPS), and disturbed shear stress. In cultured human vascular endothelial and smooth muscle cells, LPS stimulation significantly upregulated the expression of PCSK9 and lectin-like oxidized low-density lipoprotein receptor-1 (LOX-1) [24]. Similarly, TNF-α and ox-LDL also significantly induced *PCSK9* gene expression, primarily via activation of the NF-κB signaling pathway. Specifically, ox-LDL activates NF-κB through its receptors, LOX-1 and TLR4. Upon nuclear translocation, NF-κB promotes the transcription of inflammatory genes and directly binds to the PCSK9 promoter region, thereby enhancing PCSK9 expression [25]. Thus, a positive feedback loop is established within the inflammatory milieu: proinflammatory stimuli → NF-κB activation → PCSK9 upregulation → further enhancement of inflammation and ox-LDL receptor expression, ultimately amplifying the inflammatory response of ECs.

Endothelial homeostasis is preserved under conditions of physiological laminar shear stress, whereas low or disturbed flow has been shown to alter endothelial gene expression. Studies have demonstrated that exposure of human aortic ECs to low shear stress leads to elevated intracellular ROS levels and increased PCSK9 mRNA expression, whereas antioxidant treatment partially attenuates this shear-induced PCSK9 upregulation [26]. These findings suggest that hemodynamic forces play a significant role in PCSK9 regulation and may contribute to its local accumulation within atherosclerotic lesions.

PCSK9 expression is also modulated through interactions with other membrane receptor molecules. For example, a positive feedback regulatory loop exists between PCSK9 and LOX-1 in vascular cells. Under inflammatory conditions, *PCSK9* gene silencing has been shown to reduce LOX-1 expression and activity, whereas exogenous PCSK9 protein enhances LOX-1 expression. Conversely, LOX-1 knockdown downregulates PCSK9, while LOX-1 overexpression leads to PCSK9 upregulation. In mice lacking either PCSK9 or LOX-1, a significant reduction in the expression of the corresponding reciprocal protein was also observed [27]. Furthermore, mitochondrial ROS production has been identified as the initiating factor in the mutual induction of PCSK9 and LOX-1. Elevated ROS levels stimulate the expression of both proteins, whereas ROS inhibition downregulates their expression [26]. In ECs, PCSK9 expression is tightly regulated by inflammatory signaling cascades—upstream by transcription factors such as NF-κB, and downstream by positive feedback mechanisms that amplify both PCSK9 and receptor expression.

**Fig. 2.**
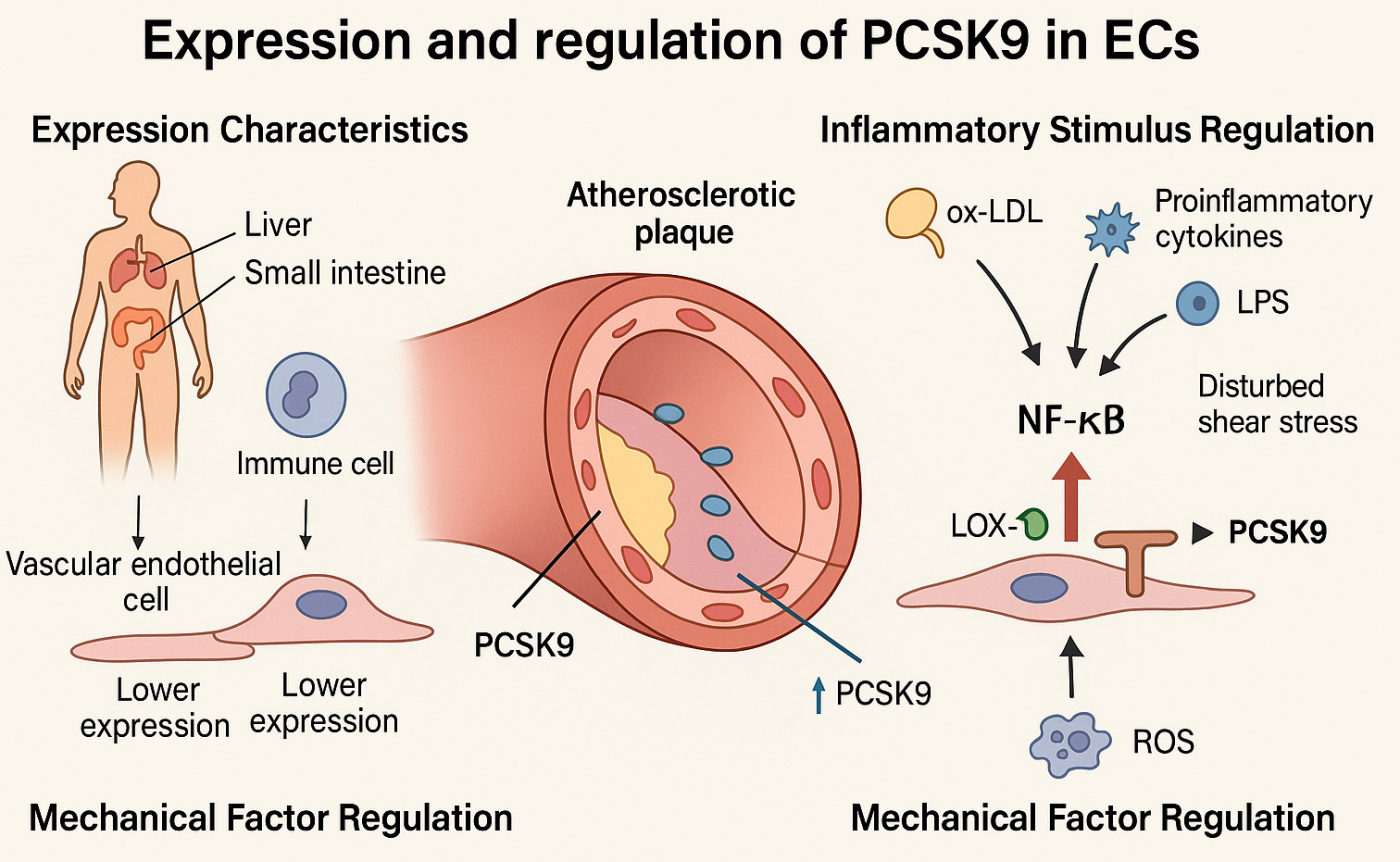
**Mechanisms regulating endothelial PCSK9 expression under atherogenic conditions**. This schematic depicts the regulatory mechanisms governing PCSK9 expression in ECs. Under homeostatic conditions, PCSK9 is predominantly synthesized by the liver and small intestine, while its expression in endothelial and immune cells remains low. Under atherosclerotic conditions, PCSK9 expression in ECs is markedly upregulated, particularly within atherosclerotic plaques. Inflammatory stimuli—including oxidized ox-LDL, proinflammatory cytokines (e.g., TNF-α, IL-1β, LPS), and disturbed shear stress—activate the NF-κB signaling pathway, thereby promoting PCSK9 transcription. ROS generated by disturbed flow further enhance PCSK9 expression and upregulate lectin-like oxidized low-density lipoprotein receptor-1 (LOX-1), establishing a positive feedback loop that amplifies endothelial inflammation and promotes atherogenesis. ox-LDL, oxidized low-density lipoprotein; TNF-α, tumor necrosis factor-α; IL-1β, interleukin-1β; LPS, lipopolysaccharide; NF-κB, nuclear factor kappa B; LOX-1, low-density lipoprotein receptor-1; ECs, endothelial cells. The figure was created with BioRender.

Overall, PCSK9 expression in ECs is modulated by multiple AS-related risk factors. It remains low under homeostatic conditions but is markedly upregulated in the presence of hyperlipidemia, proinflammatory cytokines, and disturbed shear stress. Endothelial-derived PCSK9 is subject to both systemic metabolic regulation and rapid induction by local inflammatory signals, contributing to its involvement in the pathogenesis of localized atherosclerotic lesions (Fig. 2).

## 5. Mechanistic Study of ECs-Derived PCSK9 in AS

### 5.1 Effects on the Early Stages of AS

While PCSK9 has been widely recognized for its systemic role in cholesterol metabolism via LDLR degradation, growing evidence indicates that PCSK9 also exerts critical effects during the early stages of atherogenesis, particularly in fatty streak formation and vascular smooth muscle cell (VSMC) activation [28]. These effects are mediated both by its canonical function as a secretory convertase and through LDLR-independent pathways. Recent studies have shown that PCSK9 promotes foam cell formation by enhancing the uptake of ox-LDL and impairing cholesterol efflux in macrophages. Mechanistically, PCSK9 increases the expression of scavenger receptors such as CD36 and LOX-1 while downregulating ATP-binding cassette transporter A1 (ABCA1), thereby facilitating intracellular cholesterol accumulation [29]. Notably, Shin *et al*. [14] demonstrated that PCSK9 can directly bind to the receptor CAP1 and activate TLR4 signaling, resulting in amplified oxLDL uptake and pro-inflammatory gene expression in macrophages, independent of LDLR. These findings suggest a direct pro-atherogenic role of PCSK9 in initiating foam cell formation and local inflammation at the nascent lesion site.

In addition to its effects on macrophages, PCSK9 also modulates VSMC behavior during the early atherogenic process. It promotes a phenotypic switch of VSMCs from a contractile to a synthetic state, facilitating their proliferation and migration into the intima. VSMCs not only respond to PCSK9 but also actively secrete it, establishing a feedforward loop that amplifies vascular remodeling. PCSK9-induced upregulation of inflammatory mediators such as VCAM-1 further contributes to early lesion development [29]. Furthermore, inhibition of PCSK9 has been shown to reduce VSMC-derived foam cell formation and attenuate VSMC proliferation and migration in preclinical models [30]. These data collectively support the notion that endothelial cell-derived PCSK9 may actively participate in early atherogenesis by promoting foam cell formation, initiating vascular inflammation, and driving smooth muscle cell activation. Thus, the pathophysiological role of PCSK9 extends beyond its impact on circulating lipoproteins and encompasses key cellular events that occur in the initial phases of atherosclerotic plaque formation.

### 5.2 Effects on Inflammatory Signaling Pathways in ECs

Accumulating evidence suggests that PCSK9 exerts pro-inflammatory effects by activating inflammatory signaling pathways in ECs. Elevated levels of PCSK9 have been shown to enhance the transcription of TLR4 and LOX-1. TLR4 primarily recognizes pathogen-associated molecular patterns such as LPS, while LOX-1 functions as a major receptor for ox-LDL uptake in ECs. The upregulation of these receptors facilitates the accumulation of LPS and ox-LDL in ECs, resulting in sustained activation of inflammatory pathways—particularly the NF-κB pathway, a central transcriptional regulator of pro-inflammatory cytokines and chemokines. This mechanism enables PCSK9 to establish a self-amplifying inflammatory cascade, as follows: PCSK9 elevation → upregulation of TLR4/LOX-1 → activation of NF-κB → increased inflammatory factor expression → further PCSK9 induction [31]. This mechanism extends beyond the cellular context to the atherosclerotic plaque microenvironment, where localized elevation of PCSK9 promotes inflammatory cell infiltration and activation, thereby directly accelerating plaque inflammation independently of its lipid-lowering effect. In addition to activating NF-κB signaling, accumulating evidence suggests that endothelial cell-derived PCSK9 can promote vascular inflammation via the nucleotide-binding oligomerization domain-like receptor protein 3 (NLRP3) inflammasome pathway. PCSK9 has been shown to trigger NLRP3 inflammasome assembly and caspase-1 activation, leading to the release of pro-inflammatory cytokines IL-1β and Interleukin-18 (IL-18) [32]. Consistently, silencing PCSK9 or using PCSK9 inhibitors reduces NLRP3 inflammasome activation and pyroptosis in vascular cells [33]. These findings indicate that the pro-atherogenic inflammatory effects of PCSK9 extend beyond NF-κB upregulation to involve NLRP3 inflammasome activation. Another *in vitro* study demonstrated that treatment of HUVECs with recombinant PCSK9 protein significantly upregulated the expression of VCAM-1 and ICAM-1, thereby promoting monocyte adhesion. Conversely, inhibition of PCSK9 using a monoclonal antibody or small interfering RNA (siRNA) attenuated LP-induced inflammatory responses in ECs [34]. Collectively, these findings suggest that endothelial-derived PCSK9 contributes to the amplification of local inflammatory and immune responses within atherosclerotic lesions.

### 5.3 Regulation of Apoptosis and Autophagy in ECs

PCSK9 has been shown to regulate both apoptosis and autophagy in ECs, thereby affecting atherosclerotic plaque development and stability. *In vitro*, HUVECs stimulated with 100 µg/mL ox-LDL exhibited a peak apoptosis rate at 24 hours. Concurrently, PCSK9 mRNA and protein expression levels were significantly upregulated, suggesting its involvement in ox-LDL–induced endothelial apoptosis. Upon PCSK9 knockdown in HUVECs using short hairpin RNA (shRNA), ox-LDL–induced apoptosis was markedly attenuated, as evidenced by decreased expression of the pro-apoptotic proteins Bax and caspase-3, and increased expression of the anti-apoptotic protein Bcl-2. In addition, PCSK9 knockdown inhibited ox-LDL–induced phosphorylation of the stress kinases p38 MAPK and JNK, both of which are key mediators in the MAPK signaling pathway, suggesting that PCSK9 promotes endothelial stress-induced apoptosis via MAPK activation [23]. Autophagy, on the other hand, serves as a cytoprotective mechanism under stress conditions. Ox-LDL stimulation has been shown to induce autophagy (increased LC3B-II and decreased p62) along with PCSK9 upregulation in HUVECs. Silencing of PCSK9 using shRNA further enhanced ox-LDL–induced autophagy, while attenuating endothelial damage and inflammatory cytokine release, and improving cell viability [35]. Mechanistically, PCSK9 silencing inhibited phosphorylation of the phosphoinositide 3-kinase (PI3K)/Akt/mammalian target of rapamycin (mTOR) signaling pathway, thereby relieving mTOR-mediated autophagy suppression and facilitating autophagic flux. This promoted the clearance of oxidized lipid toxicity and reduced HUVEC injury and apoptosis. These findings suggest that PCSK9 functions as a negative regulator of autophagy under oxidative stress, with its elevation suppressing autophagy via PI3K/Akt activation, while its knockdown permits enhanced autophagic activity and greater cellular resilience. Notably, PCSK9’s pro-apoptotic influence in endothelial cells may also involve classical regulators of apoptosis and the cell cycle. Recent studies indicate that endothelial PCSK9 overactivity can activate the p53 pathway, upregulating downstream targets like (cyclin-dependent kinase inhibitor 1) p21^CIP1^ and Inhibitor of CDK4a (p16^INK4a^) that enforce G_1_/S cell cycle arrest [36]. Such PCSK9-induced cell cycle arrest (senescence) is analogous to the effect of CDK4/6 inhibition, and further promotes endothelial dysfunction by predisposing cells to apoptosis. Indeed, one study showed that PCSK9 impairs cell proliferation and induces a senescent, polyploid state in vascular cells, accompanied by increased apoptosis [37]. These findings suggest that, in addition to activating stress kinases (p38 MAPK, JUN N-terminal kinases [JNK]), endothelial PCSK9 may exacerbate atherogenesis by engaging p53-mediated apoptotic pathways and cell cycle checkpoints.

Emerging evidence indicates that endothelial-derived PCSK9 can engage intracellular signaling pathways that influence autophagy and cell proliferation. For instance, PCSK9 activity has been linked to activation of the PI3K/Akt/mTOR pathway—a well-known inhibitor of autophagy—as well as the MAPK/ERK pathway, which drives cell proliferation [35]. By tipping these pathways toward a pro-growth state, PCSK9 may suppress autophagic processes in ECs. This mechanism dovetails with the concept that PCSK9’s effects align with low AMP-activated protein kinase (AMPK) activity, since active AMPK ordinarily restrains mTOR signaling to promote. Indeed, augmenting AMPK activity (e.g., via pharmacological or metabolic stimuli) has been shown to reduce PCSK9 expression and ameliorate endothelial inflammation [38]. Therefore, beyond its canonical role in LDL receptor degradation, endothelial PCSK9 might contribute to atherogenesis by concurrently promoting proliferative signaling and inhibiting autophagy. Such crosstalk between PCSK9 and pathways like Akt/mTOR and ERK not only furthers endothelial dysfunction but also suggests that therapeutically targeting PCSK9 could restore autophagic balance and mitigate excessive cell proliferation within atherosclerotic lesions [35].

In summary, PCSK9 has been shown to exert dual regulatory effects on ECs survival by promoting pro-apoptotic signaling and concurrently suppressing protective autophagic pathways, thereby accelerating atherosclerotic lesion progression.

### 5.4 Effects on Oxidative Stress in ECs

Oxidative stress is a major contributor to endothelial dysfunction and AS, and growing evidence supports a strong association between PCSK9 and endothelial oxidative stress. As previously described, ROS plays a pivotal role in the reciprocal regulation between PCSK9 and LOX-1. Binding of ox-LDL to LOX-1 induces substantial ROS generation in ECs, leading to activation of NF-κB and upregulation of PCSK9 expression. In turn, elevated PCSK9 further promotes LOX-1–mediated ox-LDL uptake, resulting in enhanced ROS production and establishing a self-perpetuating oxidative stress loop. PCSK9 may also impair the bioavailability of endothelium-derived NO. Elevated PCSK9 levels have been associated with reduced expression of endothelial nitric oxide synthase (eNOS) in aortic ECs from aged wild-type and PCSK9^-⁣/-^ mice, whereas PCSK9 inhibition has been shown to restore eNOS expression in aged arterial endothelium. Additionally, PCSK9 inhibition significantly downregulated the expression of NADPH oxidase subunits, including NOX4 and p22^phox^, in aged aortic ECs. Given that NADPH oxidase represents a major source of ROS in ECs, the observed downregulation of NOX4 suggests attenuated endothelial oxidative stress [39]. PCSK9 and AMPK Inactivation: Emerging evidence indicates that endothelial PCSK9 can impair AMP-activated protein kinase (AMPK) signaling, which in turn promotes oxidative stress. Ox-LDL exposure upregulates PCSK9 in vascular ECs while concurrently reducing phosphorylated AMPK levels, suggesting PCSK9 is associated with AMPK inactivation. Conversely, interventions that activate AMPK (e.g., the exercise-hormone irisin) suppress PCSK9 expression and vascular inflammation, highlighting an inverse relationship between AMPK activity and PCSK9. Meanwhile, irisin restored p-AMPK in ox-LDL–treated ECs and downregulated PCSK9, alleviating oxidative damage [38]. These findings support that PCSK9 impairs the AMPK pathway, removing AMPK’s protective effects against oxidative stress (AMPK normally activates antioxidant defenses and inhibits NADPH oxidase and NF-κB inflammation. Consistently, clinical data indicate that inhibition of PCSK9 exerts cardioprotective effects partly via AMPK reactivation, improving mitochondrial biogenesis and reducing oxidative injury [40]. Taken together, endothelial PCSK9 may contribute to oxidative stress by inactivating AMPK, thereby diminishing AMPK’s antioxidant functions. These findings suggest that elevated PCSK9 levels lead to heightened oxidative stress in ECs, whereas PCSK9 inhibition restores redox homeostasis, enhances NO production, and improves endothelial function. Additionally, endothelial-derived PCSK9 may regulate oxidative stress and inflammation through modulation of the anti-aging molecule sirtuin 1 (SIRT1). It has been proposed that excessive PCSK9 suppresses SIRT1 activity in ECs, thereby attenuating its antioxidant protective functions and promoting premature cellular senescence. Conversely, PCSK9 inhibition enhances SIRT1 activity, thereby reducing oxidative stress and the expression of cellular senescence markers [36]. Although the precise mechanism remains to be elucidated, it is hypothesized that PCSK9 disrupts the balance between antioxidant defenses and oxidative stress generation in ECs, thereby increasing their vulnerability to oxidative injury. In conclusion, PCSK9 contributes to increased oxidative stress in ECs and facilitates AS progression by enhancing ox-LDL uptake and activating inflammatory signaling pathways.

### 5.5 Effects on Vascular Permeability and Endothelial Barrier Function

Impairment of the endothelial barrier is a critical determinant of AS development. The integrity of this barrier can be compromised by PCSK9, thereby increasing vascular permeability through multiple mechanisms. First, PCSK9-mediated inflammatory activation upregulates the adhesion molecules ICAM-1 and VCAM-1 on ECs surfaces, thereby promoting leukocyte adhesion and transmigration and reflecting diminished tight-junction integrity and heightened barrier permeability. Inhibition of PCSK9 reduces ICAM-1 expression and monocyte infiltration into the endothelium, consequently improving EC function. Second, PCSK9-induced upregulation of LOX-1 increases ox-LDL retention within ECs, initiating a cascade that includes ROS production and NF-κB activation. These events disrupt cytoskeletal organization and tight-junction architecture, ultimately elevating endothelial permeability [9]. Thus, PCSK9 exacerbates barrier disruption by amplifying the ox-LDL/LOX-1 signaling axis. Finally, PCSK9-induced endothelial apoptosis and detachment disrupt the endothelial monolayer and erode plaque fibrous caps, permitting blood components to penetrate plaque cores and further compromising the barrier.

Collectively, PCSK9 disrupts intercellular junctions and impairs reparative mechanisms via inflammatory and apoptotic pathways, ultimately compromising barrier integrity and increasing vascular permeability to lipids and inflammatory cells. This disruption is particularly detrimental in AS because impaired barrier function facilitates the translocation of atherogenic substances into the vessel wall, thereby accelerating plaque development and destabilization. Therefore, modulating PCSK9 expression in ECs may preserve or restore endothelial barrier integrity and thus provide therapeutic benefit against AS in Table 1 (Ref. [23, 31, 34, 35, 36, 39]).

**Table 1.** **Mechanisms by which endothelial cell-derived PCSK9 contributes to AS**.

Mechanism	Model used	Authors	Ref.
PCSK9 upregulates TLR4 and LOX-1 in ECs, enhancing ox-LDL and LPS uptake and activating NF-κB-mediated inflammatory cascades; PCSK9 enhances LOX-1-mediated ox-LDL uptake and ROS production, forming a ROS–LOX-1–PCSK9 positive feedback loop.	ECs (*in vitro*)	Ding Z e*t al*.	[31]
Recombinant PCSK9 increases VCAM-1/ICAM-1 expression and monocyte adhesion; PCSK9 inhibition reduces LPS-induced inflammation; PCSK9 increases ICAM-1/VCAM-1 expression and monocyte adhesion, weakens cell junctions, leading to higher vascular permeability.	HUVECs (*in vitro*)	Leung AKK *et al*.	[34]
PCSK9 expression is increased in ox-LDL-treated ECs, promotes apoptosis via Bax/caspase-3 and p38 MAPK/JNK pathways; silencing PCSK9 reduces apoptosis; PCSK9-induced endothelial apoptosis contributes to endothelial erosion and barrier dysfunction, facilitating lipid infiltration.	HUVECs (*in vitro*)	Li J *et al*.	[23]
PCSK9 suppresses protective autophagy via PI3K/Akt/mTOR; knockdown enhances autophagy, reduces inflammation, and improves EC survival.	HUVECs (*in vitro*)	Li W *et al*.	[35]
PCSK9 reduces eNOS and increases NOX4/p22phox expression, impairing NO bioavailability and increasing oxidative stress in aging ECs.	Aortic ECs from aged mice	Liu S *et al*.	[39]
PCSK9 inhibits SIRT1 expression, increasing oxidative stress and senescence markers; inhibition of PCSK9 activates SIRT1 pathway.	HUVECs (*in vitro*)	Wang Y *et al*.	[36]

TLR4, Toll-like receptor 4; ox-LDL, oxidized LDL; VCAM-1, vascular cell adhesion molecule-1; ICAM-1, intercellular adhesion molecule-1; PI3K/Akt/mTOR, phosphoinositide 3-kinase /Akt/mammalian target of rapamycin; eNOS, endothelial nitric oxide synthase; NO, nitric oxide; SIRT1, anti-aging molecule sirtuin 1; HUVECs, human umbilical vein endothelial cells; p38 MAPK/JNK, p38 mitogen-activated protein ki- nase/JUN N-terminal kinases; NOX4/p22, nicotinamide adenine dinucleotide phosphate oxidase 4/p22.

## 6. Intervention Strategies Targeting Endothelial PCSK9

Given the potential significance of endothelial PCSK9 in AS, multiple future therapeutic strategies are being actively explored:

**RNA-based interventions**, including RNA interference and gene editing, may offer direct suppression of PCSK9 expression in ECs. For instance, siRNA and antisense oligonucleotides (ASOs) have been developed to selectively silence the *PCSK9* gene. Systemically administered siRNAs—such as inclisiran—are already in clinical use to suppress hepatic PCSK9 production and reduce circulating cholesterol levels. In the future, siRNAs could be engineered for targeted endothelial delivery by conjugation to aptamers or antibodies specific for endothelial adhesion molecules. Such approaches may enrich siRNA uptake in ECs, allowing for localized PCSK9 knockdown. This strategy may enable both early prevention of AS and targeted intervention at lesion sites, complementing systemic lipid-lowering effects.

**Local delivery of PCSK9-neutralizing monoclonal antibodies** is another promising avenue. Current antibody therapies require subcutaneous or intravenous administration and systemic circulation. Local administration could improve bioavailability at lesion sites while reducing systemic exposure. For example, anti-PCSK9 antibodies could be coated on stents for sustained local release at vulnerable plaques, or directly delivered via interventional catheter into diseased arterial segments. This approach aims to neutralize both circulating PCSK9 and locally secreted PCSK9 from endothelial cells or macrophages within the plaque microenvironment, thereby enhancing plaque stabilization and preventing disease progression. The feasibility of this approach could initially be evaluated in preclinical models by assessing parameters such as plaque inflammation reduction and fibrous cap thickening.

**Endothelial-targeted nanocarriers** represent an emerging platform for precision drug delivery. Nanoparticles encapsulating PCSK9 inhibitors (e.g., small molecules, peptides, or nucleic acids) can be functionalized with targeting ligands—such as VCAM-1 antibodies or thrombin peptide motifs—that recognize activated endothelium. These ligand-modified carriers can preferentially adhere to inflamed ECs and release cargo locally at atherosclerotic sites. Preclinical studies have demonstrated effective targeted delivery of therapeutic agents to injured endothelium using nanoparticles conjugated with cRGD peptides [41]. Therefore, incorporating anti-PCSK9 molecules into such targeted systems may yield dual benefits—lipid-lowering and anti-inflammatory effects—while reducing systemic adverse reactions.

**Other innovative strategies** include CRISPR-Cas9–based gene editing to permanently knock out PCSK9 at lesion sites, bispecific antibodies to simultaneously inhibit PCSK9 and LOX-1, and agents that activate protective endothelial signaling pathways to counteract PCSK9-induced dysfunction. While these approaches remain largely at the proof-of-concept stage, continued advancements in gene editing and nanomedicine are expected to facilitate their translation.

It must be emphasized, however, that topical or cell-specific targeting of PCSK9 must be approached with caution. Complete PCSK9 inhibition may disrupt endothelial repair or smooth muscle homeostasis. Therefore, precise dosing, spatial targeting, and safety assessment are crucial. The immunogenicity and metabolic clearance of targeted delivery systems must also be thoroughly evaluated. In summary, endothelial PCSK9 represents a compelling therapeutic target in AS, and further progress in delivery technologies and preclinical validation is essential to pave the way toward clinical application.

## 7. Conclusions and Perspectives

AS is a complex pathological condition driven by both systemic and local factors. Recent studies on PCSK9 have significantly advanced our understanding of lipid metabolism, leading to the development of transformative lipid-lowering therapies. However, growing evidence suggests that the role of PCSK9 extends beyond cholesterol regulation, contributing to atherosclerotic plaque formation through its effects on vascular wall cell biology, particularly on ECs. Studies summarized in this review indicate that PCSK9 derived from ECs may contribute to AS pathogenesis by promoting inflammation, increasing vascular permeability, and inducing apoptosis and cellular senescence. This local effect appears to be partially independent of the systemic lipid-lowering actions of PCSK9, underscoring its potential as a novel therapeutic target in AS.

Despite significant progress, several critical knowledge gaps and unresolved questions persist in current research efforts. (1) Mechanistic understanding: The molecular mechanisms underlying PCSK9 activity within ECs require further elucidation. For instance, it remains unclear which specific endothelial receptors or signaling molecules interact directly with PCSK9. These questions warrant investigation through protein interaction studies and signaling pathway analyses. (2) Quantitative contribution: The relative contribution of endothelial-derived PCSK9 to overall AS remains uncertain. Since circulating and endothelial-localized PCSK9 are challenging to distinguish experimentally, their respective roles must be clarified using refined models, such as endothelial cell-specific PCSK9 knockout mice. (3) Clinical validation: Current understanding of endothelial PCSK9 remains largely experimental, with limited validation in human studies. Future efforts may consider assessing endothelial PCSK9 expression in vascular biopsy or endarterectomy specimens from patients with AS and examining its correlation with plaque inflammation and stability. In addition, advanced imaging modalities may be employed to evaluate whether PCSK9 inhibition influences plaque characteristics, such as inflammatory burden or fibrous cap thickness, thereby providing evidence of its localized effect. (4) Therapeutic strategy balance: Interventions targeting endothelial PCSK9 must carefully account for potential off-target effects, including impacts on hepatic lipid metabolism and systemic immunity. Achieving local therapeutic effects without disrupting systemic functions remains a key challenge and requires careful evaluation in large-animal models or clinical trials.

In conclusion, the investigation of endothelial-derived PCSK9 has expanded current insights into the pathogenesis of AS, offering novel perspectives for both preventive and therapeutic strategies. With the development of emerging interventions—such as vaccines, gene therapy, and nanomedicine-based delivery platforms—it is anticipated that both the lipid-regulatory and local pro-inflammatory roles of PCSK9 can be simultaneously targeted. This dual-targeting strategy may enable a more comprehensive and effective management of AS, not only by reducing circulating cholesterol levels, but also by improving the vascular inflammatory microenvironment and endothelial function. Consequently, plaque progression and subsequent cardiovascular events may be more effectively prevented. The continued integration of fundamental and translational research will be essential to resolve outstanding scientific questions and to facilitate the translation of laboratory findings into innovative, patient-centered therapies.
